# Supplementary material for: Artificial Intelligence-Enabled Electrocardiography Detects B-Type Natriuretic Peptide and N-Terminal Pro-Brain Natriuretic Peptide
Source: Diagnostics (Basel). 2023 Aug 22;13(17):2723. doi: 10.3390/diagnostics13172723 (PMC10487184; doi:10.3390/diagnostics13172723)
Supplement: Supplementary file 1 [file diagnostics-13-02723-s001.zip › diagnostics-2549357-supplementary.pdf]

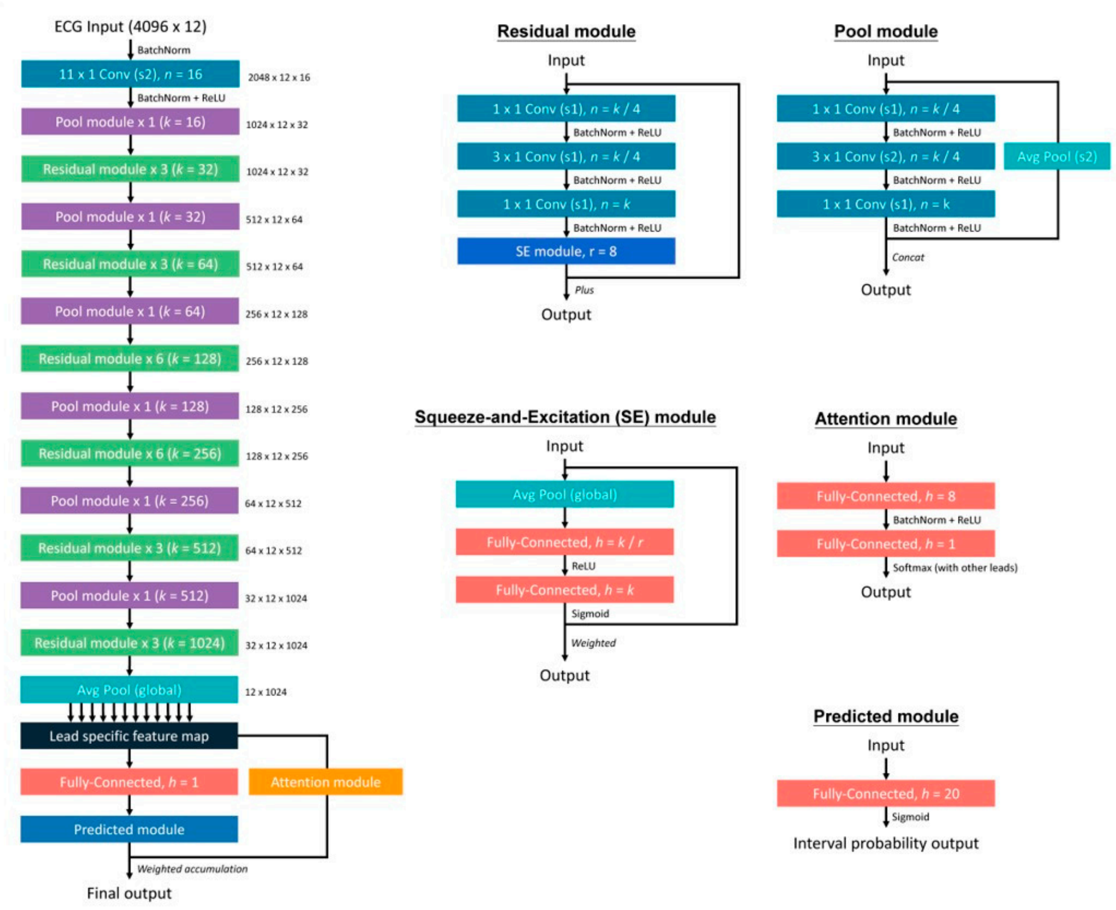

Figure S1. The implementation of our deep learning model. The model architectures of the deep learning model for analyzing ECG.
